# The participatory and partisan impacts of mandatory vote-by-mail

**DOI:** 10.1126/sciadv.abc7685

**Published:** 2020-08-26

**Authors:** Michael Barber, John B. Holbein

**Affiliations:** 1 Department of Political Science, Brigham Young University, 745 Kimball Tower, Provo, UT 84602, USA.; 2 Batten School of Leadership and Public Policy, University of Virginia, 111 Garrett Hall, Charlottesville, VA 22903, USA.

## Abstract

Recently, mandatory vote-by-mail has received a great deal of attention as a means of administering elections in the United States. However, policy-makers disagree on the merits of this approach. Many of these debates hinge on whether mandatory vote-by-mail advantages one political party over the other. Using a unique pairing of historical county-level data that covers the past three decades and more than 40 million voting records from the two states that have conducted a staggered rollout of mandatory vote-by-mail (Washington and Utah), we use several methods for causal inference to show that mandatory vote-by-mail slightly increases voter turnout but has no effect on election outcomes at various levels of government. Our results find meaning given contemporary debates about the merits of mandatory vote-by-mail. Mandatory vote-by-mail ensures that citizens are given a safe means of casting their ballot while simultaneously not advantaging one political party over the other.

## Teaser

This paper shows that mandatory vote-by-mail increases voter turnout but does not advantage one political party over the other.

## INTRODUCTION

With the recent coronavirus disease 2019 (COVID-19) outbreak, mandatory vote-by-mail (hereafter VBM) and its close variants (e.g., no-excuse absentee voting) have received a great deal of attention as a means of administering elections in the United States. Many experts have suggested that VBM would allow elections to proceed while simultaneously minimizing the spread of the highly contagious and deadly virus. As a result, some states (e.g., Hawaii, Illinois, Vermont, and Nevada) have recently passed standby legislation that would transition their elections to all-mail by mailing ballots to all of their citizens if the COVID outbreak continues or worsens, while other states have moved partially in this direction by opting to send absentee ballot applications to all registered voters (e.g., Arizona and Idaho), and others still have made decisions to loosen restrictions for obtaining mail-in ballots (e.g., Massachusetts, New Hampshire, and Texas). (For a thorough overview of these recent changes and their various iterations, see “State Voting Policy Changes to Deal with COVID-19,” the National Vote at Home Institute.) Beyond these handful of states, many other local, state, and even federal policy-makers have publicly and prominently debated making changes to move toward all-mail voting; both nominees for president have spoken widely on the merits of mandatory VBM, too many legislators to mention have gone back and forth on the merits of all-mail elections, and numerous activist groups (e.g., the American Civil Liberties Union, Action Network, and FreedomWorks) have a move toward a universal VBM system. Many of these debates hinge crucially on whether mandatory VBM advantages one party over the other. For instance, President Trump and other Republicans have repeatedly railed against variants of VBM. Consequentially, the debate over the merits of this electoral reform has become contentious and highly polarized. Recent polls have found that while more than 8 in 10 Democrats supported all-mail elections, only 4 in 10 Republicans held the same position (*1*). Many assume, act as if, or even directly argue that VBM will substantially advantage Democrats at the ballot box.

What is the effect of mandatory VBM on electoral outcomes in the United States? Existing research has studied the effects of VBM (mandatory and voluntary) on overall levels of voter turnout (*2*–*11*) and on the turnout levels of demographic subgroups broken down by age, gender, and race (*4*, *12*, *13*). However, previous VBM studies have tended to only look at effects in individual states, not at scale nationwide. Moreover, no published work has looked at whether VBM affects partisan election results. As noted elections expert C. Stewart succinctly puts it, “[E]vidence so far on which party benefits [from VBM has] been inconclusive” (*14*, *15*).

Here, we use a unique combination of historical nationwide county-level data from the past three decades (1992–2018) and more than 40 million individual-level voter records from two states (Washington and Utah) paired with various methods for causal inference to estimate the effect of mandatory VBM on voter turnout and election outcomes. We show that VBM has a modest positive effect on turnout, but it has no measurable effect on how well Democratic candidates perform at the ballot box. VBM could offer an opportunity to, at worst, maintain historical levels of turnout or, at best, even slightly increase low levels of turnout while simultaneously not substantively advantaging one political party over the other.

## DATA

**Fig. 1:**
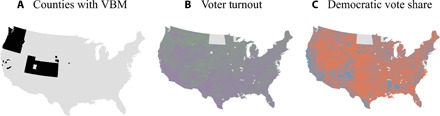
Mandatory VBM (**A**), voter turnout (**B**), and Democratic vote share (**C**) in 2018.

Here, our key treatment variable is an indicator for whether or not a county conducted a general election entirely (or overwhelmingly in some cases) via mail-in ballots. Several states and counties within states have adopted this method of election administration for federal elections—California (five counties, 2018), Oregon (all since 2000), Washington (staggered, 1996–2012), Utah (staggered, 2012–2020), Colorado (all since 2014), and Nebraska (four counties, 2018). Although these systems of VBM have differences of administration, they are all consistent in the core elements of mandatory VBM in that they (i) mail all constituents a ballot in the lead-up to Election Day and (ii) limit or omit in-person voting. Consistent with many other studies of the effect of election laws (which often have slight variations in administration), we estimate average treatment effects of the variants of how VBM is administered. This quantity is of direct relevance to states that are considering the typical experience of states that already implemented this reform. [To go one step further, however, in the Supplementary Materials, we explore whether subtle differences in the administration of VBM (e.g., the presence or absence of vote centers and the state-wide adoption or adoption only in a few counties) influence our estimates. They do not appear to do so; see figs. S7 and S8, and their surrounding discussion, in the Supplementary Materials.] Because of the spatial and temporal variation in moving to VBM (see Fig. 1 for current levels and figs. S1 to S3 for change over time), we can leverage various statistical methods to estimate a causal effect of conducting an election via VBM.

Our key outcome measures are voter turnout and partisan vote margins. We use three data sources to calculate these values. We examine turnout given that previous work in this space has theorized (and provide evidence to support the fact) that VBM may increase the number of people who vote by informing them of their right to vote (through the mailed communication/ballot from the government) and making that process more convenient (*8*–*10*). The first is turnout numbers and party vote shares at the county level over the past three decades (1992–2018) from Dave Leip’s Atlas of Elections, which is widely used in social science research. The Leip dataset provides a county-level measure of votes cast and how the two political parties perform in U.S. elections over time. The second source is the U.S. Census Bureau, which provides measures of total county population (1992–2018) and citizen voting age population (CVAP, 2004–2018). Together, the Leip and Census data produce turnout rates and partisan vote shares for each county in each election cycle. The resultant dataset is composed of just over 42,000 county-year observations. In this dataset, our two dependent variables are turnout rates in the county-year and two-party Democratic vote shares in races for the House, Senate, governorship, and presidency, as well as an average of these races in that county and year. We use an index measure as one of our outcomes to avoid idiosyncrasies of any particular election (i.e., candidate specific characteristics, issues specific to the race, local factors, etc.) and to reduce residual noise.

Figure 1 displays the current state of VBM as well as turnout levels and Democratic vote share in the most recent federal election (2018). In Fig. 1B, green indicates higher levels of turnout (purple is lower). In Fig. 1C, blue indicates areas where Democrats do better, whereas red indicates the opposite. Maps from 1994 to 2018 can be found in the Supplementary Materials (figs. S1 to S3). (North Dakota is omitted from the analysis as data on the timing of the rollout of VBM at the local level are not readily available and this state has a somewhat distinct set of election laws from other states.)

Our third data source comes from 40 million voting records from the states of Washington and Utah; these data have been collated by the data and analytics firm DT Client Services LLC. In the United States, whether a citizen vote (but not who they vote for) is public record, voter files contain voting and registration histories of all registered voters in the state. Although registration records are publicly available in all states, states vary in how much information the file provides. Effectively all states provide registered citizens’ vote history, age, gender, address, political party, and name (to list just a few). We have voting data in Utah spanning from 2012 to 2018 and voting data in Washington spanning from 2002 to 2016.

We focus on Washington and Utah as these are the only two states that have gone from little mandatory VBM to full implementation of mandatory VBM, with counties staggering their implementation, in recent years. (As noted earlier, a few counties in California and Nebraska implemented mandatory VBM in 2018. However, the recency of the change and the relatively small penetration of this reform do not allow us to use individual voter turnout records.) With Washington and Utah data, we can examine the effects of VBM on overall turnout and turnout by a voter’s political party, which has direct implications for the partisan impacts/nonimpacts of VBM (given low rates of crossover voting). As we describe in the next section, using individual-level data from Utah and Washington has trade-offs, but drilling down into these states is very useful given that doing so allows us to improve the internal validity and precision of our estimates compared to those derived from aggregate-level data alone.

## METHODS

Our empirical approach here is a difference-in-differences design. The main assumption of a difference-in-differences model is the parallel trends assumption, which asserts that, in the absence of treatment, the potential outcomes of the treated and the potential outcomes of the untreated observations run parallel over time (*16*).

The most common approach of implementing a difference-in-differences design in the study election laws (and in difference-in-differences designs more generally) is the two-way fixed effects model (*17*). As its name implies, this model includes unit (county or state) and time (year) fixed effects. This approach is outlined in Eq. 1, where *V_ct_* represents the treatment of interest [whether a county (*c*) has mandatory voting in a given year (*t*)]. *O_ct_* represents the outcomes we explore (turnout and vote share), and α*_t_* and γ*_c_* represent year and county fixed effects, respectively. This model specification does not account for factors that vary across units over time.

$$O_{\mathrm{ct}}=\beta_{0}+\beta_{1}V_{\mathrm{ct}}+\alpha_{t}+\gamma_{c}+\epsilon_{\mathrm{ct}}$$ (1)

Although this is the most common approach to estimating a difference-in-differences model, there are econometric reasons why a two-way fixed effects design may be insufficient for identifying the causal effect of mandatory VBM. First, scholars have recently used proofs and simulations and applied examples to show that two-way fixed effects often struggle to obtain the causal effect of interest (*18*–*20*). Second, in the case of mandatory VBM, there are reasons to move beyond this specification. A standard check in the difference-in-differences literature involves looking for treatment effects on outcomes before treatment has occurred (*20*). When we run this specification (fig. S10), we find signs of imbalance in the lagged dependent variables. That is to say, a two-way fixed effects model would lead us to conclude that mandatory VBM increased voter turnout/Democratic vote share even before it was put into law. Since this is definitely not the case, this instead suggests that the two-way fixed effects model may be biased and that the model is violating the parallel trends assumption. If we rely on this modeling approach, any effects we observe may be driven by pretreatment imbalances in our outcomes. [We also note that the standard state (instead of county) and year fixed effects model shows similar signs of pretreatment imbalance.]

Given this concern, our preferred difference-in-differences models consist of an extension of Eq. 1 that includes county, state-by-year fixed effects, and individual time trends for each county. This is a standard recommendation in the difference-in-differences literature, especially when the two-way fixed effects models fail to produce desired levels of pretreatment balance (*20*, *21*), as is the case with mandatory VBM. The models with linear county-specific time trends are displayed in Eq. 2. This model absorbs all observed and unobserved factors that remain constant within counties (e.g., political culture, social capital, and rigid political institutions) and that are shared within years and states (e.g., recessions, specific candidates on the ballot, and differential campaign investments) and trends that vary across counties (e.g., the natural trends of voter turnout, partisan vote shares, and other factors in counties). (Our results are robust to using a quadratic county-specific time trend; see fig. S11.)

$$O_{\mathrm{ct}}=\beta_{0}+\beta_{1}V_{\mathrm{ct}}+\alpha_{\mathrm{st}}+\gamma_{c}+\sigma c*t+\epsilon_{\mathrm{ct}}$$ (2)

The virtue of the model estimated in Eq. 2 is that it allows for better causal identification. The inclusion of county-specific time trends (σ*c* * *t*) allows us to relax the tenuous parallel trends assumption key to difference-in-differences specifications. Here, our identifying assumption is that our outcomes deviate from county-year effects by following the trend captured by the interaction of time with each county. Under this assumption, identification comes from sharp deviations from otherwise smooth county-specific trends. The assumptions behind this approach are considered to be less strict than those required in a model with only unit and time fixed effects (*16*, *22*). This fact bears out in the mandatory VBM case. The same specification tests that we use with the two-way fixed effects model show balance on our lagged outcomes when we include county time trends (see fig. S10). In other words, once we take into account the temporal trends of our outcomes across counties, the treatment (VBM implementation) no longer predicts turnout before the implementation of the treatment. This suggests that this model is better situated to identify the causal effect of mandatory VBM. For this reason, in our county-level analyses, models with county fixed effects, state-by-year fixed effects, and county-specific time trends are our preferred estimates. Given the desirable properties of this model, we present results from models that take the form outlined in Eq. 2.

In addition to the approach that we outlined here, we also run a host of other robustness checks, which (for the sake of space) we include in the Supplementary Materials. These further tweak various aspects of the difference-in-differences design and include using different versions of fixed effects (see fig. S5), focusing on just those that implemented county-by-county staggered VBM versus states that adopted mandatory VBM statewide (see fig. S7), coding VBM as varying degrees of treatment that take into account slight differences in the types of VBM policies that states implement (see fig. S8), and omitting one treatment state at a time to ensure that our results are not driven by a single outlier state (see fig. S6). In all cases, these models yield similar results to those discussed here (i.e., slight increases in turnout with no advantage for Democrats at the ballot box). Last, we look at whether the presence of mandatory VBM in one’s county is related to voter registration. If mandatory VBM is unrelated to registration patterns, we are unlikely to have an issue with differential registration bias. Figure S4 shows that VBM is unrelated to registration rates.

In addition to these robustness checks, we implement a second identification strategy that leverages individual-level voter registration data from two states: Utah and Washington. These are the only two states that have implemented VBM in a staggered fashion in recent years for which voter file data are available.

We implement this second identification strategy for two reasons. First, it allows us to improve our statistical precision. When presenting a series of null findings (as we do below regarding Democratic vote shares), we need to be sure that we are not treating any nonsignificant effects as evidence of no effect—a common mistake often made in empirical research that finds null effects (*23*). Instead, we want to pay attention to how wide our confidence intervals are, to give a sense of the types of effect sizes we can rule out. Individual-level data allow us to make very precise inferences using equivalence testing of what types of effects we can and cannot rule out (*23*, *24*). Although our data consist of individuals nested in counties (which we adjust for by clustering SEs at the level of treatment), we still gain a great deal of statistical power and precision above and beyond analyses that only use aggregate data. [Clustered SEs inflate SEs proportional to the number of clusters and observations within clusters (*25*, *26*). Given that the penalty clustered SEs apply is not as harsh as collapsing nested data to the level of the treatment, we would expect our estimates of individual-level, nested data to be more precise than estimates at the aggregated county level. However, our results are robust to collapsing the voter file to the county level and clustering at an even more conservative level; see fig. S12.]

The second, and perhaps more important, reason that we use individual-level voter file data is that it allows us to improve our ability to draw causal inferences even further than the aggregated data allow. Even with pretreatment balance, difference-in-differences models may be biased (*19*). Thus, the individual-level models provide additional robustness to our county-level analysis.

In short, the gains to internal validity and precision from using individual-level data are vitally important given the policy relevance of the effects of VBM and given that, to convincingly argue that VBM has no partisan impacts, one has to narrow their confidence intervals as much as possible to minimize the possibility of type 2 error. Given the current political terrain, getting the best causal identification and highest degree of precision is paramount.

Using individual-level data allows us to leverage individual-level changes in exposure to mandatory VBM. Our approach uses individual-voter fixed effects. This identifies the effect of mandatory VBM based only on variation in the system that individual voters live in (either because they moved or because their county changed its voting system). That is to say, it estimates the effect of an individual seeing a change to whether the place that they live has mandatory VBM or not (above and beyond natural changes from one election cycle to the next, which are accounted for in the year fixed effects included in the model).

These models control for all observable and unobservable individual-level heterogeneity that remains constant within individuals over time (e.g., individual-level propensity to vote, family-level propensity to vote, genetics, childhood experiences, stable personality traits, political motivation, family background, political upbringing, etc.). This provides a very stringent robustness check in exploring the relationship between mandatory VBM in one’s community and individual turnout. The approach is particularly strong and is, therefore, often used in contexts (such as ours) where randomization of the treatment is not readily available (*27*–*29*). With these models, we look for the effect of VBM on turnout of individuals of various political parties. This has direct relevance to the potential effects on party vote shares given historically low rates of crossover voting in the United States (*30*, *31*).

While this modeling approach is especially powerful at purging bias, it does come with a drawback in that it is limited to the two states wherein we can conduct our analysis. However, pairing Utah and Washington together allows us to draw estimates from two meaningful contexts—one where (according to the Cooperative Congressional Election Study) a majority of voters identify or lean Republican (Utah, 52.6%) and one where a majority of voters identify or lean Democrat (Washington, 50.3%). While these two states do not mirror national averages on all dimensions and are unique in their own ways, we note that this is true of any analysis that leverages single states or subsets of states. Studies that dive into richer data within single/multiple states must grapple with the trade-off between the benefits to internal validity/precision that comes at the expense of external validity. In our case, however, we argue that what we may lose in external validity by focusing on these two states alone (of necessity given our identification strategy), we gain in internal validity.

With this additional identification strategy, we are able to make our estimates as robust as possible using observational data. In addition, even though Utah and Washington are unique, our results from these states confirm the findings in the aggregate-level data that include all states. Doing so gives a more comprehensive picture of the effects of VBM than either analysis on its own would provide. We note that for this very reason, it is common in election law studies [for example, those exploring the effects of preregistration (*32*) and same-day registration (*33*)] to use nationwide data paired with analyses that then drill down to individual states.

Together, our analyses leverage data from several sources and multiple methods for causal inference. They provide rich insights by using both individual and aggregate level data. Given the many methods and datasets that we use, our paper provides the most thorough and comprehensive look at the causal effects of mandatory VBM on voter turnout and election outcomes to date. Its scope goes beyond that of previous papers that have focused only on overall turnout rates. In addition, our methods provide a clearer picture of VBM’s effects than analyses based only on geographically aggregated data.

## RESULTS

**Fig. 2:**
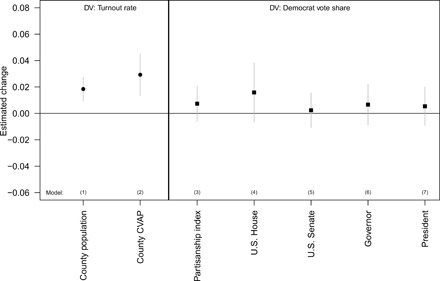
Effects of VBM on voter turnout and election results in the United States. Dv, dependent variable.

Figure 2 shows the results in two panels—first, the effect on overall turnout (using total county population in the left estimate and the CVAP in the right), and second, the effect on Democratic Party vote shares. All models are ordinary least squares with county and state-by-year fixed effects with individual county time trends (see Eq. 2). All in all, Fig. 2 provides evidence from seven different model specifications (two for turnout and five for party vote share). These results, combined with the additional robustness checks that we run in the Supplementary Materials, ensures that the effects we are estimating are robust.

Looking at the left panel of Fig. 2, we see that across both model specifications, VBM has a modest effect on aggregate levels of voter turnout. This effect is robust. Depending on the specification, the effects range from 1.8 (model 1, *P* < 0.001) to 2.9 percentage points (model 2, *P* < 0.001). These estimates are consistent with previous research on the aggregate turnout effects of VBM, which have generally found small to modest effects of VBM (*2*–*7*). However, our results are important in that they show that VBM’s turnout effects are present even at scale. This finding is important given that previous studies have tended only to examine individual states. It has a vital meaning given current policy debates, which focus on implementing mandatory VBM nationwide.

How large or small are the effects of VBM on turnout relative to other interventions designed to increase voter turnout? These estimated effects are roughly equivalent to somewhere between one nonpartisan get-out-the-vote solicitation over the phone and one social-pressure mailer (*34*). Moreover, when considering substantive significance, it is important to put these turnout effects into the current context. In the midst of a pandemic, we must shift our counterfactual. That VBM increases turnout moderately suggests that (at worst) it can be a viable stand-in for in-person voting during the COVID-19 pandemic. This possibililty is important because many convenience voting reforms that move citizens away from in-person social interaction (such as early in-person voting) have been shown to actually decrease turnout (*35*). That mandatory VBM sees modestly higher levels of voter turnout than in-person voting even though it decreases in-person interaction is a testament to this policy’s effectiveness (especially in the context of the COVID-19 outbreak).

Does VBM influence who wins elections? All of our models indicate that mandatory VBM has no meaningful effect on how well Democrats do in elections. Our most precise estimates (model 3) suggest that VBM increases Democratic vote shares by 0.7 percentage points; however, the 95% confidence interval extends from −0.7 percentage points to 2 percentage points. Despite having a great deal of statistical power, these effects are not close to statistically significant (*P* = 0.29) and are substantively small. Even if we completely ignore the statistical uncertainty around our estimates (we think that it is unwise to do so), however, this suggests that VBM could only matter in the rarest of cases. For context, only 1.5% of our county observations in our dataset have an electoral margin this narrow. This is a small fraction of counties that influences even a smaller number of races at the state and federal level. Moreover, it is important to remember that despite having high statistical power, this effect is not statistically distinct from a zero (or even a small advantageous effect for Republicans) effect and it is very precisely estimated—we can confidently rule out effects as small as a 2.09 percentage point gains in favor of Democrats. In short, VBM does not have modest or even large effects on Democratic candidates’ performance in elections.

**Fig. 3:**
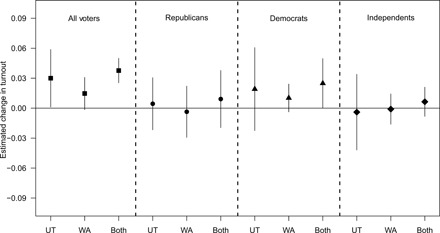
Effects of VBM on voter turnout among partisan subgroups in Washington and Utah.

Figure 3 displays results from individual-level voter file data in Utah and Washington with individual, county-year linear time trends, and year fixed effects (robustness checks for this dataset are shown in fig. S11). While the overall turnout effects (i.e., all groups pooled) are modest and statistically significant, none of the effects across Republicans, Democrats, or Independents are statistically significant at traditional levels. However, the main point of interest in Fig. 3 is the fact that VBM has consistent effects across subgroups; Republicans, Democrats, and Independents see similar, statistically indistinguishable effects. In all models in Fig. 3, the differences in effects among these groups is not statistically significant. For example, the *P* value for the difference between the coefficients estimated in model 6 (Republicans in the pooled sample) and model 9 (Democrats in the pooled sample) is 0.41: not statistically significant despite a higher degree of statistical power. This result suggests that even when we account for the many observed and unobserved factors that are constant within individuals themselves, mandatory VBM has precisely no effect on which political party will perform well in elections.

Some may wonder how VBM can increase voter turnout but not advantage one political party over the other, given the conventional wisdom that nonvoters skew toward the Democratic Party. In response to this question, we think that it is important to note that our result is consistent with other election law studies that show that easing voting/registration restrictions increases turnout but has no effect on electoral outcomes (*32*, *36*). This conclusion is also consistent with more general research that suggests that despite the fact that nonvoters tend to lean more democratic as a whole, this gap is smaller than you might expect and, as such, increasing voter turnout does not necessarily advantage one party over the other (*37*, *38*). This may have something to do with the types of voters that VBM mobilizes. To us, it seems possible, perhaps even likely, that VBM is pulling in individuals who are on the fence about voting, of which there are plenty of individuals in both political parties. In making voting marginally easier, it does not cater to the pool of voters who fundamentally are not interested in politics or in voting. The fact that the turnout effects VBM produces are small to modest in size likely contributes to VBM not advantaging one party over the other.

In sum, across a myriad of model specifications provided here (plus 30 additional models in the Supplementary Materials), three different datasets that span the last three decades, and two different identification strategies, we provide the most precise and robust evidence to date that shows that, after accounting for factors that are unrelated to VBM itself, this reform modestly increases turnout but has no effect on who wins elections.

## DISCUSSION

Mandatory VBM increases turnout modestly in general elections but does not substantively advantage either political party. These results are vitally important given contemporary debates at local, state, and federal levels over the merits of this mode of administering elections. They have special meaning given that many governments are currently considering how to proceed with the 2020 (and beyond) elections in the midst of the COVID-19 outbreak. They have direct relevance to states that have introduced legislation that would allow mandatory VBM (e.g., Illinois, Vermont, and Nevada) and also to the many other states that are currently debating the extent to which they should use mail-in voting to conduct elections moving forward.

We note that while VBM’s effect on turnout is modest, the counterfactual one uses matters a great deal. In elections—like the present one—where citizens have to choose between minimizing the chances that they contract or spread COVID-19 and fulfilling their civic duty to vote, levels of voter participation could likely stagnate, decline, and/or become more unequal than they already are. Given this possibility, allowing citizens to cast their ballots from the safety of their own homes is a viable approach to ensuring that elections continue despite the deadly COVID-19 pandemic.

In short, mandatory VBM preserves public safety while also maintaining the current balance of power between the two dominant political parties. VBM preserves both public health and the integrity of elections.

## Supplementary Material

abc7865_SM.pdf

## SUPPLEMENTARY MATERIALS

Supplementary material for this article is available at http://advances.sciencemag.org/cgi/content/full/6/35/eabc7685/DC1
