# Supplementary material for: The participatory and partisan impacts of mandatory vote-by-mail
Source: Sci Adv. 2020 Aug 26;6(35):eabc7685. doi: 10.1126/sciadv.abc7685 (PMC7449689; doi:10.1126/sciadv.abc7685)
Supplement: abc7865_SM.pdf [file abc7865_SM.pdf]

Science Advances
AAAS

advances.sciencemag.org/cgi/content/full/6/35/eabc7685/DC1

Supplementary Materials for

The participatory and partisan impacts of mandatory vote-by-mail

Michael Barber* and John B. Holbein*

*Corresponding author. Email: barber@byu.edu (M.B.); jh5ak@virginia.edu (J.B.H.)

Published 26 August 2020, *Sci. Adv.* **6**, eabc7685 (2020)
DOI: 10.1126/sciadv.abc7685

This PDF file includes:

Supplementary Text
Figs. S1 to S13
References

1 Additional Research on the Effects of Vote-by-Mail

Given space constraints in the main paper, we cannot outline in great detail studies that have been done in the past on the effects of vote-by-mail. Suffice it to say, however, a rich literature in political science has studied vote-by-mail, leveraging key insights about how making this reform available to voters influences them and the broader political world around them. While we cannot cite/outline all studies on vote-by-mail, here we briefly expand on some of the influential published pieces in this literature to provide context for our study.

Early studies provide a thorough overview of the particulars of VBM (*39–44*). The literature on vote-by-mail has considered issues of implementation and security (*45–48*). It has come up with many innovative ideas for on-the ground administration and research on the challenges and hurdles that voters (*49*), campaigns (*50–53*), and election administrators face (*48, 54–56*).

In one of the earlier works on VBM, Magleby uses data from Berkeley, California to look at differences in turnout rates across jurisdictions that had VBM and those that did not (*7*). He also looks by race, age, income and education. His identification strategy is a conditional-on-observables approach, wherein he controls for observable features of the voter/electorate. Southwell and Burchett likewise conditions on observable factors when looking at different jurisdictions with Oregon (*43*). They find that vote-by-mail is strongly related with turnout in Oregon, "second only to the impact of a presidential contest" (72. However, Gronke and Miller challenge this result (*57, 58*) (for other studies in Oregon, see (*41, 42, 59–63*)). Other studies in Oregon have concluded that those who utilize optional vote-by-mail tend to be older, more urban, and less partisan (*64*) (for other studies exploring observable differences in usage rates of optional VBM, see (*5, 12, 13, 60, 65*)). In a nice summary piece that considers many of the broader consequences of vote-by-mail systems, Charles Stewart provides a comprehensive analysis of the costs and benefits of this reform (*39*).

Menger et al. study the adoption of vote-by-mail in Colorado (*8, 10*). They find that "VBM elections lead to greater ballot completion, but that this effect is only substantial in presidential elections" (pp. 1039). As a part of their study on the effects of voting technology on voter turnout in California, Alvarez et al. explore the relationship between optional vote-by-mail and the residual vote rate in the state (*15*) (for another study of California's system, see (*66*)). They find that "regardless of the election, increased use of the mail to cast ballots is robustly associated with a significant rise in the residual vote rate" (pp. 658). Holbein and Hillygus use difference-in-differences models look at the effects of optional vote-by-mail on youth turnout, finding that it has small effects on this subgroup (*36*). Kousser and Mullin use matching-on-observables with data from Oregon and California and find that "voting by mail does not deliver on the promise of greater participation in general elections (pp. 428)" (*3*). Recent work has shown that vote-by-mail may also increase political discussion and information acquisition (*65, 67*).

Finally, political scientists have considered how campaigns and various get-out-the-vote (GOTV) organizations would interface with a VBM system. In a series of GOTV experiments, Arceneaux, Kousser, and Mullin show that "door-to-door mobilization campaigns have a larger effect on the participation of those who vote at polling places than on registrants assigned to cast mail ballots, but only among individuals whose voting behavior is most likely to be shaped by extrinsic social rewards (pp. 882)" (*50*). Other GOTV experiments in a vote-by-mail context have tested the efficacy of various voter contact strategies see (*51–53*), generally finding the standard set of get-out-the-vote interventions can still operate reasonably well in a vote-by-mail system.

In short, our work takes an important step in this literature in that it is the largest and most comprehensive study of mandatory vote-by-mail to date and takes the important step of honing in on differential effects by political party (in large-scale individual-level voter files) and on

the effects on party vote shares. While research in political science has studied vote-by-mail for decades, our work builds on the previous literature in important ways. Most previous studies take a ‘conditional-on-observables’ approach rather than approaches that utilize exogenous variation in VBM. Moreover, many previous works take a single state approach, with many focusing on Oregon or California or Colorado, but not pooling these together. And only a few exceptions use rich, large-scale, individual-level voter files in the analysis of vote-by-mail; many rely on self-reported surveys which may have issues with social desirability in the outcome measure and sampling framework, with many surveys not being designed and simply being under-powered to drill down into the dynamics at a sub-state level (*68*). They also tend to focus on overall turnout rates, usually only look by demographic characteristics other than political party. Furthermore, many studies only explore optional vote-by-mail (i.e. no excuse absentee voting) rather than all mail/mandatory vote-by mail. Our work takes an important step forward going beyond a conditional-on-observables designs and leveraging exogenous variation in vote-by-mail over time, pooling across multiple states to improve our levels of external validity, leveraging rich individual-level voter file data to increases our statistical precision and ability to draw causal inferences, focusing on turnout rates across political parties, bringing party vote shares into the analysis, and by drawing attention to the (currently actively debated) role of mandatory/all mail voting instead of optional vote-by-mail.

2 Adoption of Mandatory Vote-by-Mail Over Time

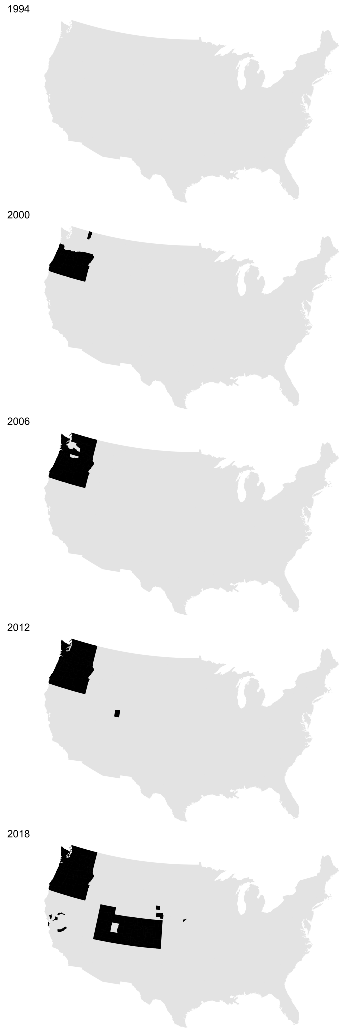

Figure S1: Adoption of Mandatory Vote-by-Mail Over Time - Maps display the progressive rollout of mandatory vote-by-mail across counties and states within the United states from 1994-2018.

3 Trends in Voter Turnout Over Time Mapped

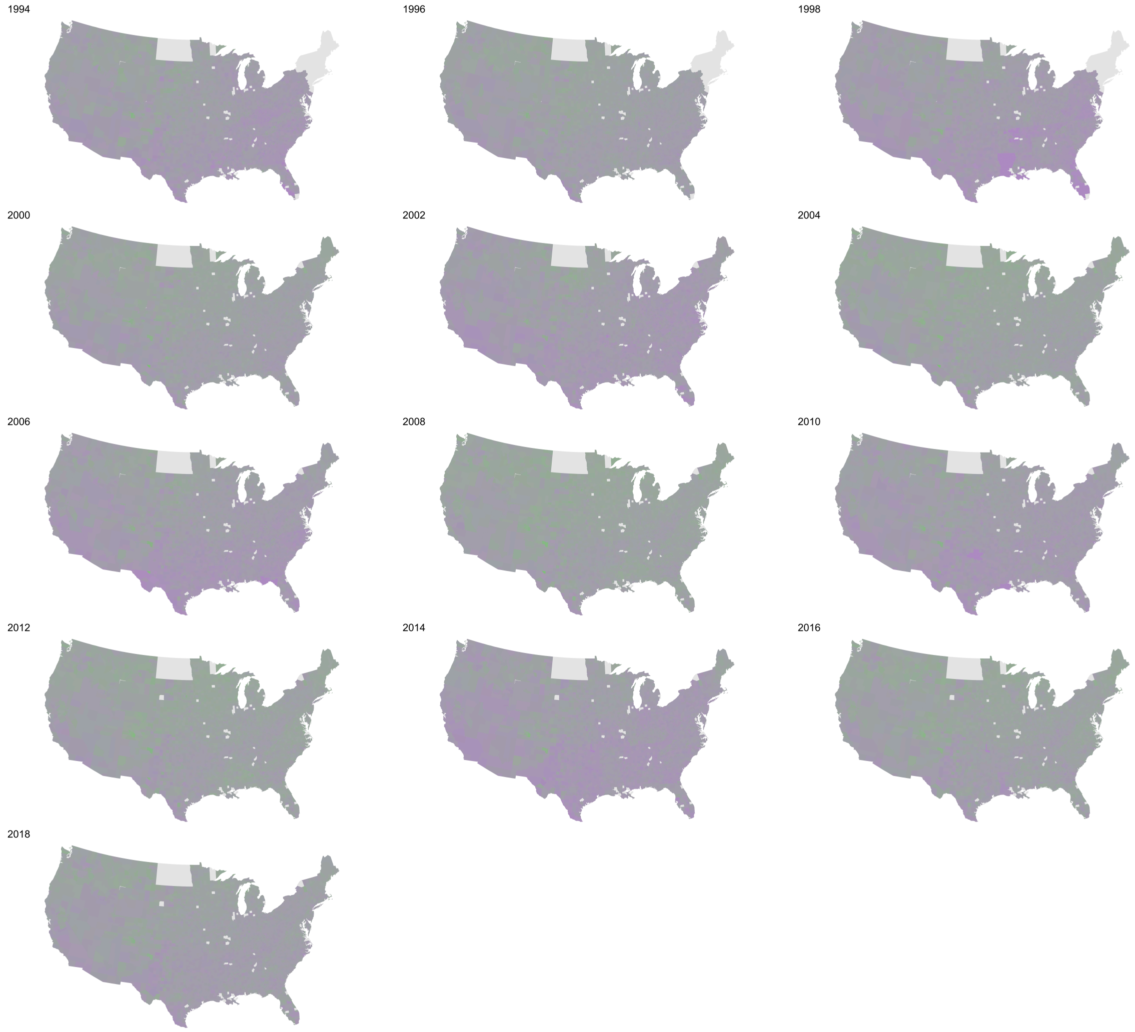

Figure S2: Voter Turnout Over Time Mapped - Maps display the voter turnout at the county-level from 1994-2018. Green indicates higher levels of voter turnout, whereas purple indicates lower levels. North Dakota is omitted from the analysis, as data on the timing of the rollout of vote-by-mail is not readily available. Several states in New England are not included in the Leip turnout files from 1994-1998; the results are robust to simply omitting these years from our analysis.

4 Trends in Democratic Vote Share Over Time Mapped

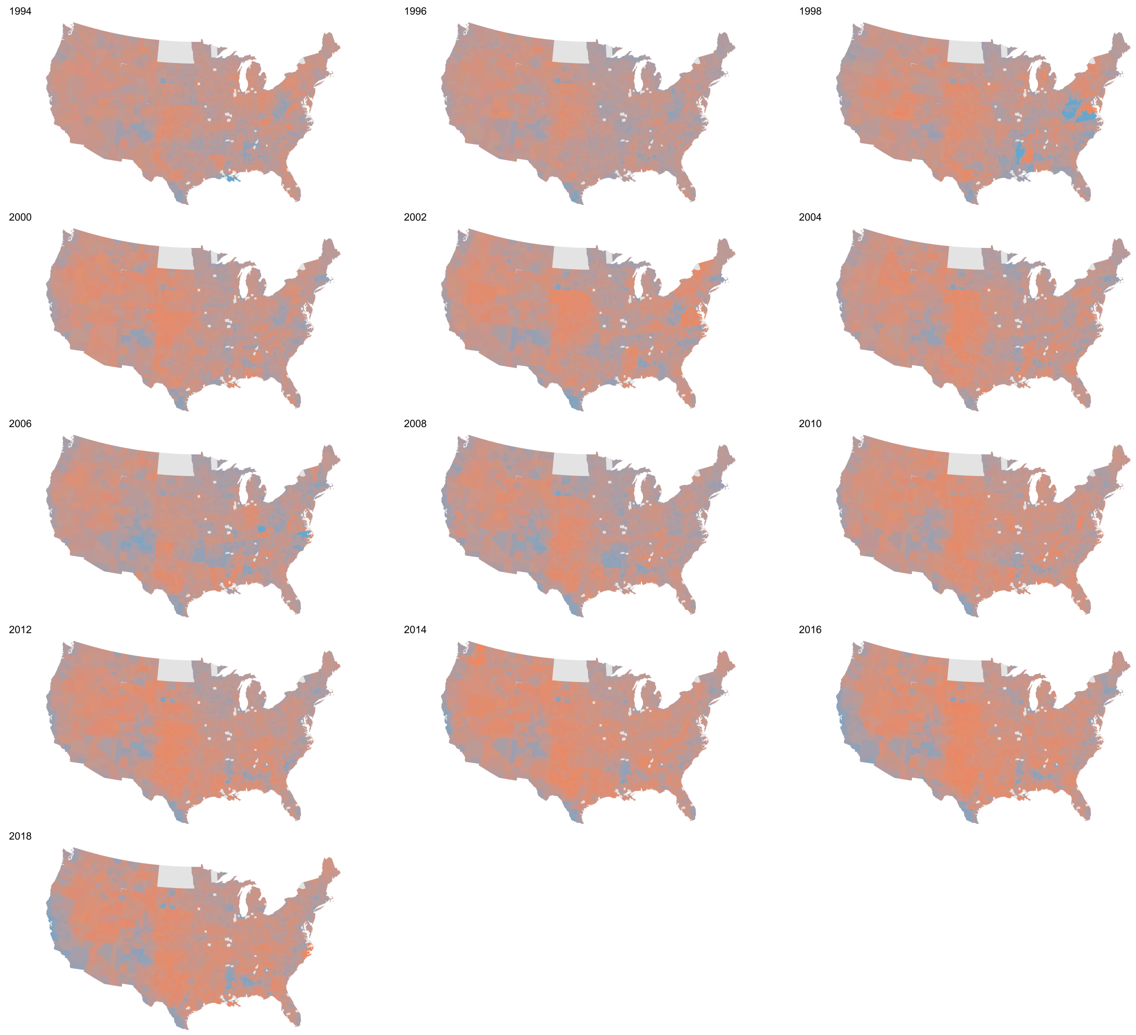

Figure S3: Democratic Vote Share Over Time Mapped - Maps display the democratic vote share at the county-level from 1994-2018. Blue indicates areas where Democrats do better, whereas red indicates the opposite. North Dakota is omitted from the analysis, as data on the timing of the rollout of vote-by-mail is not readily available.

5 Are the Results With Voter Files Confounded by Differential Registration Bias? Effects of Mandatory Vote-by-Mail on Rates of Voter Registration

One potential concern with looking only among those registered to vote—as we have to do in the voter registration lists—is that it may bias our conclusions, particularly in the regression models we run given the potential for differential registration bias (*69*). Exploring this possibility is not possible using voter files alone. However, we can test for this possibility by using data from one of the most-commonly used sources in political science—the Cooperative Congressional Election Study (CCES). Here, we look at whether the presence of mandatory vote-by-mail in one's county is related to voter *registration* as measured by the CCES (which matches participants to voter file data from another voter file vendor Catalist). If mandatory vote-by-mail is unrelated to registration patterns, we are unlikely to have an issue with differential registration bias.

Figure S4 shows this test. It plots effects across a number of specifications listed in the note of the figure. As can be seen in the Figure, mandatory vote-by-mail in one's community has little effect on whether an individual chooses to register or not. Despite being well-powered, the estimate is not close to being significant and equivalence testing allows us to rule out effects that are very small (*23, 24*). This suggests that our approach in the Utah and Washington analyses is appropriate.

Figure S4: Effects of Mandatory Vote-by-Mail on Rates of Voter Registration

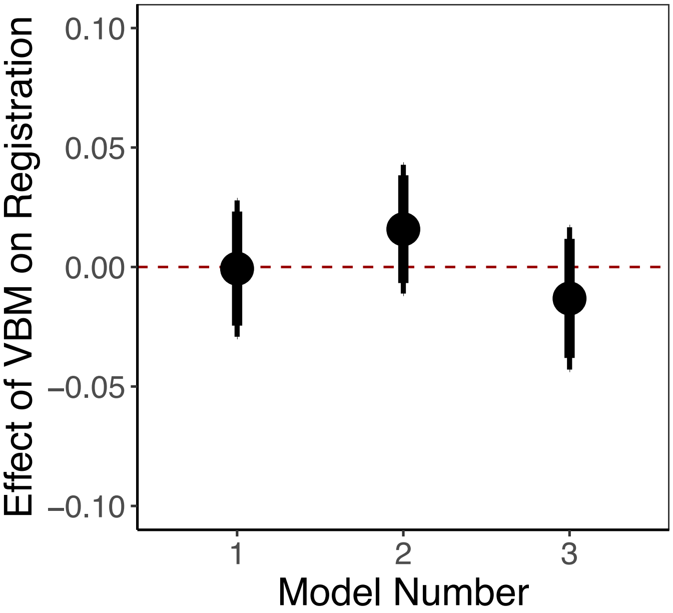

Note: Regression results from the Cooperative Congressional Election Study (CCES) 2006-2008 samples. The models estimate the effect of mandatory vote-by-mail in one's county (the lowest geographic level available in the CCES) and individual-level registration (from the CCES). Model 1 includes state and year fixed effects and a linear state-specific time trend. Model 2 includes county and year fixed effects. Model 3 includes county and year fixed effects plus a a linear state-specific time trend. The models all include controls for age, race, education, income, ideology, and political interest. The takeaway point from this figure is the presence of mandatory vote-by-mail does not affect the chances an individual registers to vote, hence differential registration bias is unlikely in our Utah-specific analysis.

6 Additional Robustness Checks

Figure S5 presents results using a variety of modelling strategies indicated on the x-axis. The first type of model includes only county and year fixed effects, the most common approach of implementing a difference-in-differences design in the study election laws (*17*). This approach is outlined in Equation (1) of the main paper. Importantly, this model does not account for factors that vary across units over time, and Section 7 below shows that this model is subject to endogeneity bias. The second type of model includes county and state-by-year fixed effects, which partially address the endogeneity concerns but still requires the parallel trends assumption to hold. The third type of model in Figure S5 includes county and state-by-year fixed effects as well as state-specific time trends. This modelling approach relaxes the parallel trends assumption, but only at the state level. As discussed in the main paper, our preferred model includes county-specific time trends to allow for differential temporal trends by county, particularly since individual counties within states adopted VBM at different times in four of the six states.

Figure S5: Alternative Modelling Strategies

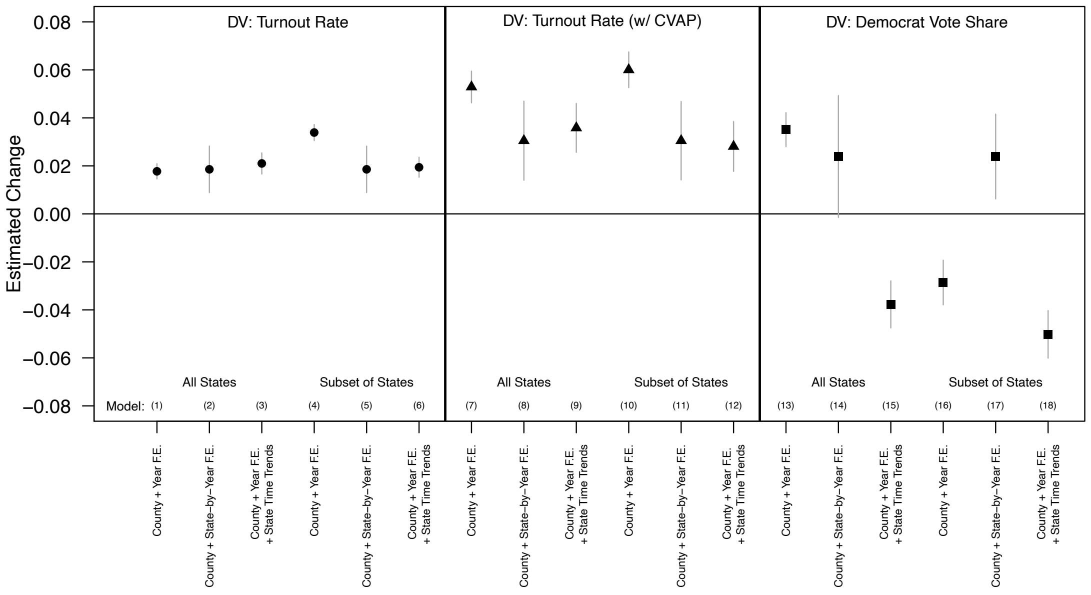

Note: Coefficient plot testing different modelling strategies. Subset estimates (fourth through sixth in each panel) include only the six states that eventually use VBM in some counties (WA, OR, CA, UT, CO, NE). Turnout effects are robust across model specifications. Partisan effects vary depending on modelling specification. Standard errors are clustered at the county-year level.

Are the results being driven by a particular state? Figure S6 reruns the county-level analysis omitting one state at a time (labeled on the x-axis if the figure). Here we use our preferred model specification that includes county and state-by-year fixed effects as well as linear county time trends. As can be seen, the results are robust to iteratively holding out one state at a time. The results are quite stable—with all not statistically significant and able to rule out very precise estimates.

Figure S6: Omitting One State at a Time

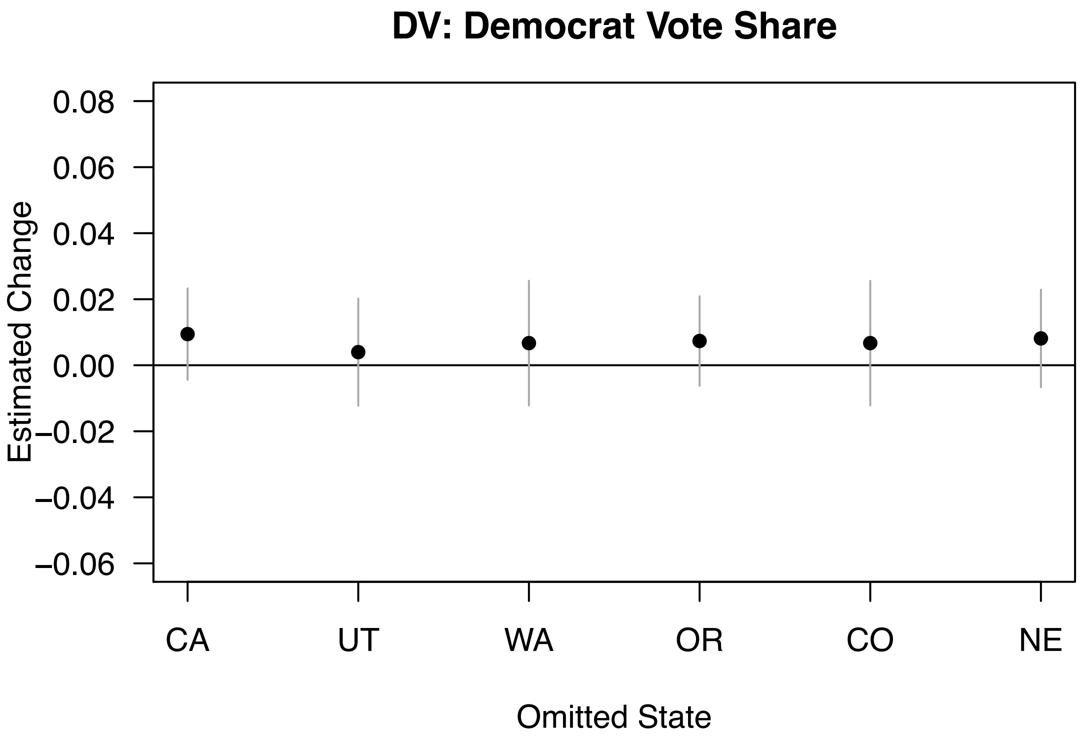

Note: Coefficient plot testing omitting one treated state at a time. Points are coefficient estimates and bars are 95% confidence intervals.

Figure S7 does something similar to Figure S6, but here we hold out states where the entire state adopted at the same time (OR, CO), as opposed to a county-by-county rollout (CA, UT, WA, NE). As the right panel shows, our results become even more precise and remain not significant. This should not be surprising as these are the counties providing identification for the nationwide models that all include county-level fixed effects.

An alternate approach that we can take is to focus exclusively on states that adopt VBM all at once and compare the change in voter turnout in these states to places that did not adopt. Unfortunately, this approach is inherently noisy given that we have only two treated units. The left panel in Figure S7 shows that, unsurprisingly, in this setup the estimates are highly sensitive to model specification. A simple two-way fixed effects design would suggest that VBM increases democratic vote share by a full 4 percentage points. However, the confidence intervals for this estimate are very wide. Once we add in state-specific time trends, this effect goes away and the effect is now negative. This sensitivity is consistent with the literature on difference-in-differences designs wherein there are few treated units. In these case, the estimates may be highly unreliable. However, our results using county implementation are robust and when we include the state-wide adopters the effect is the same. Estimating the effect of statewide adoption when there are only two treated units is inherently much more difficult than estimating the effect of county-level staggered rollout.

Figure S7: Statewide Adoption vs. County-by-County Adopters

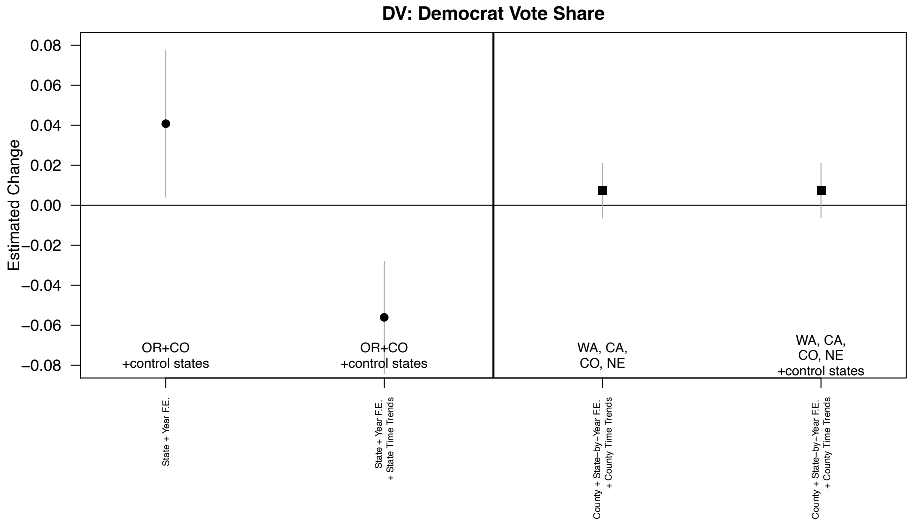

Note: Coefficient plot testing for whether the type of adoption (be it county-by-county or statewide) influences our results. Points are coefficient estimates and bars are 95% confidence intervals.

Figure S8 differentiates between mandatory vote-by-mail in states that also maintain vote centers (CA, CO) and those that do not (UT, WA, OR, NE). To do so, we code the treatment variable in two ways. First, we include an indicator variable for both kinds of VBM (right side of the panel) and second we construct a continuous measure of ease in using the vote-by-mail system: 0 (no VBM), 1 (VBM with vote centers), and 2 (only VBM). These values allow us to account for various types of VBM exposure. As can be seen, either way we code this variable the results are the same—VBM does not affect democratic vote share. fi

Figure S8: Comparing Full VBM versus VBM with Vote Centers

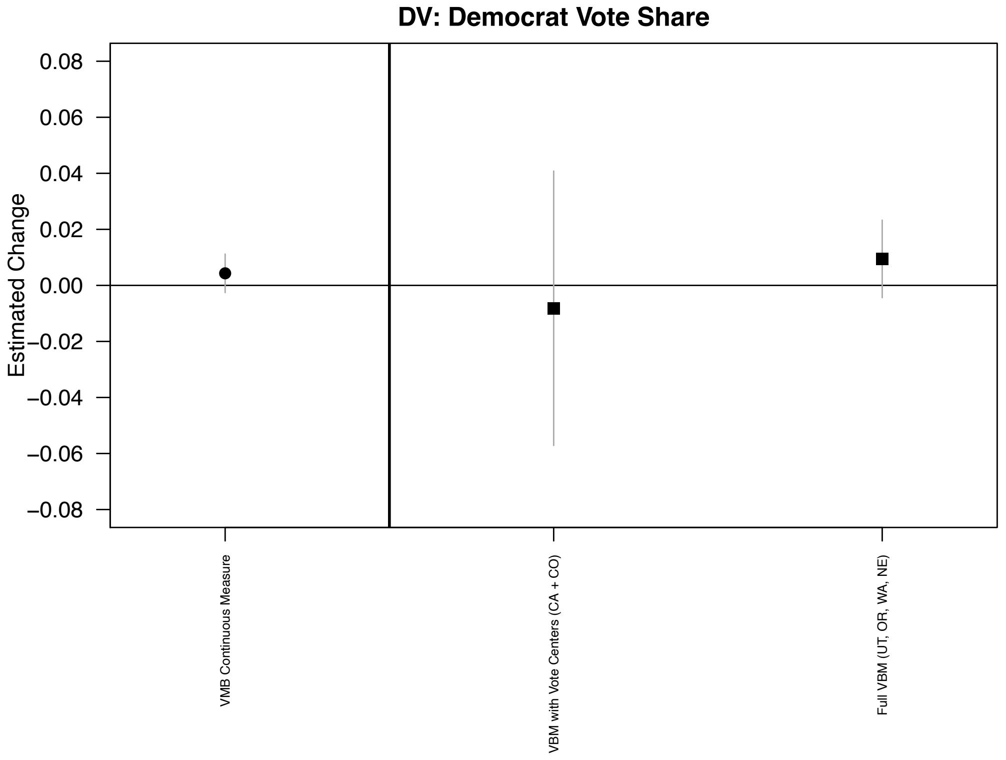

Note: Coefficient plot testing for effects when coding VBM in different ways that account for VBM with and without vote centers. Points are coefficient estimates and bars are 95% confidence intervals.

Figure S9 splits apart our measure of partisan vote shares into the four separate component election types - U.S. House, U.S. Senate, Governor, and Presidential elections. We see that the results are the same across all four types of elections—adoption of VBM does not increase democratic candidates' vote shares up and down the ballot.

Figure S9: Different Election Results Run Separately

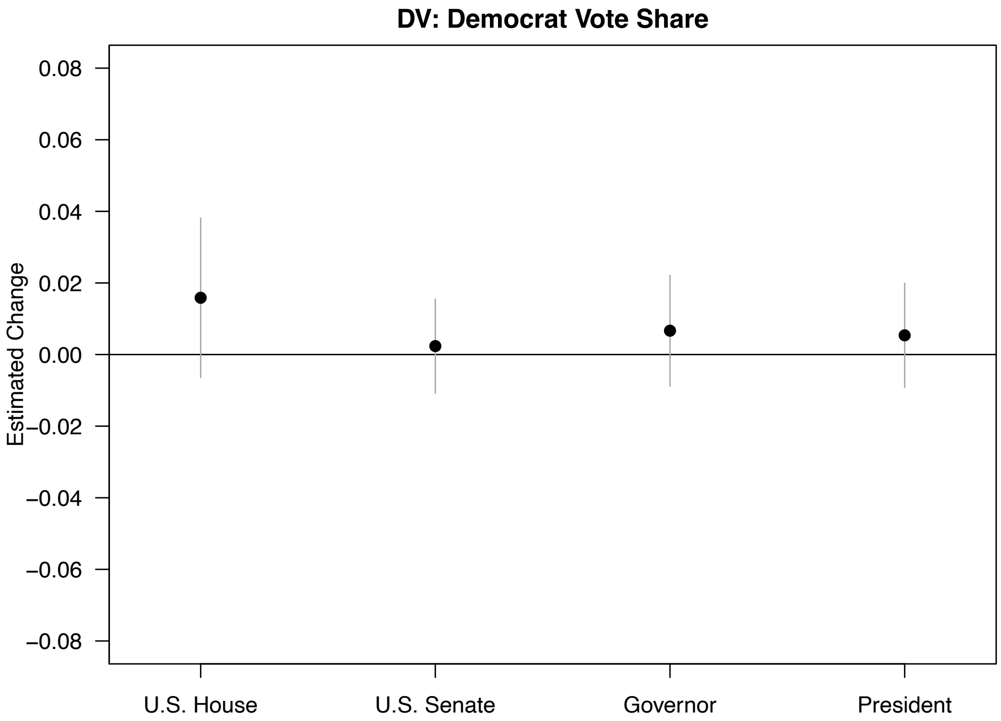

Note: Coefficient plot testing different elections separately rather than averaged together (as is shown in the main paper).

7 Testing the Parallel Trends Assumption

Figure S10 tests whether current-year VBM adoption predicts *previous-year* turnout and vote share. If this is the case, this suggests a violation fo the parallel trends assumption that is key to the validity of the difference-in-difference design. The two-way fixed effects models show signs of imbalance. However, adding county time trends improves balance considerably, making models with these trends preferential to a two-way fixed effects model without county time trends.

Figure S10: Testing for Lagged DV Effects

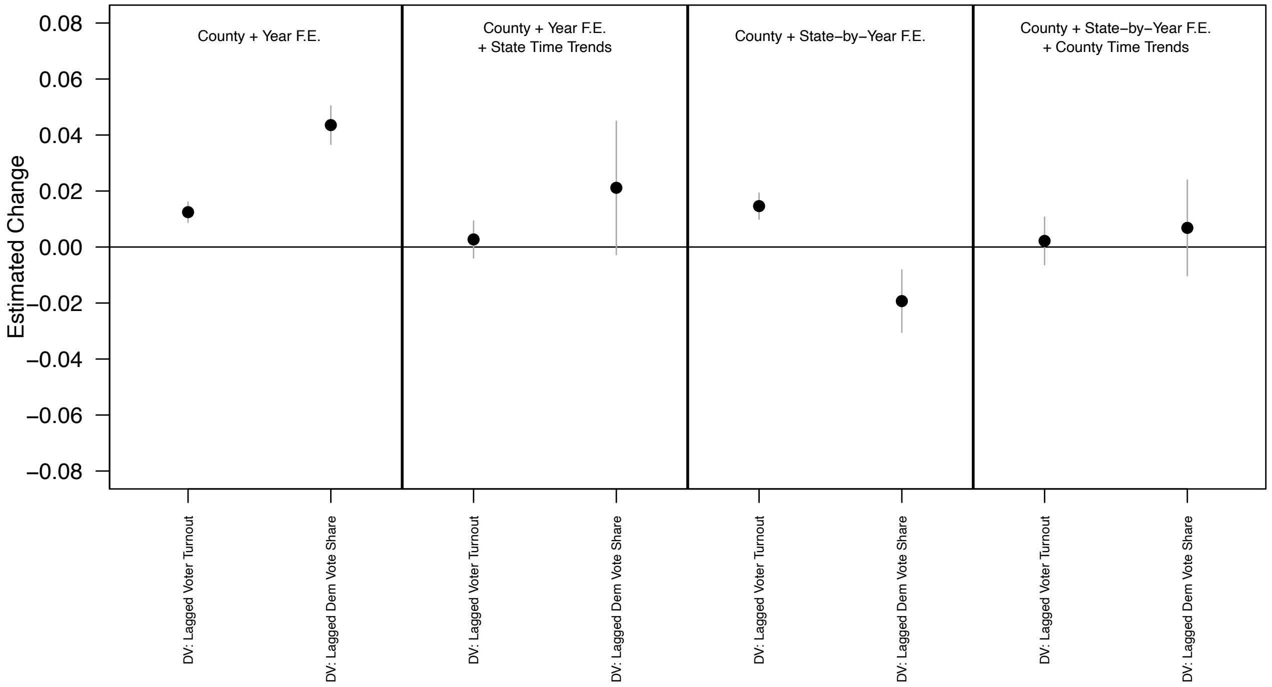

Note: Coefficient plot testing for effects on lagged outcomes across two model specifications (facets). Points are coefficient estimates and bars are 95% confidence intervals.

8 Additional Robustness Checks in the Voter File Data

Figure S11 provides additional results from the individual voter file analyses in Utah and Washington. To do so, we run two additional specifications—the first with only individual and year fixed effects and the second with individual fixed effects, year fixed effects, and a quadratic county-specific time trend. We runs these models for Utah and Washington alone and then the two states together. As can be seen, across all specifications there is an indistinguishable effect of VBM implementation on turnout among Republican, Democratic, or Independent voters. The p-values for the test of the difference between Republican and Democrat coefficients from left to right in the figure are: 0.55, 0.67, 0.35, 0.43, 0.42, and 0.49.

Figure S11: Additional Specifications with the Voter File Data

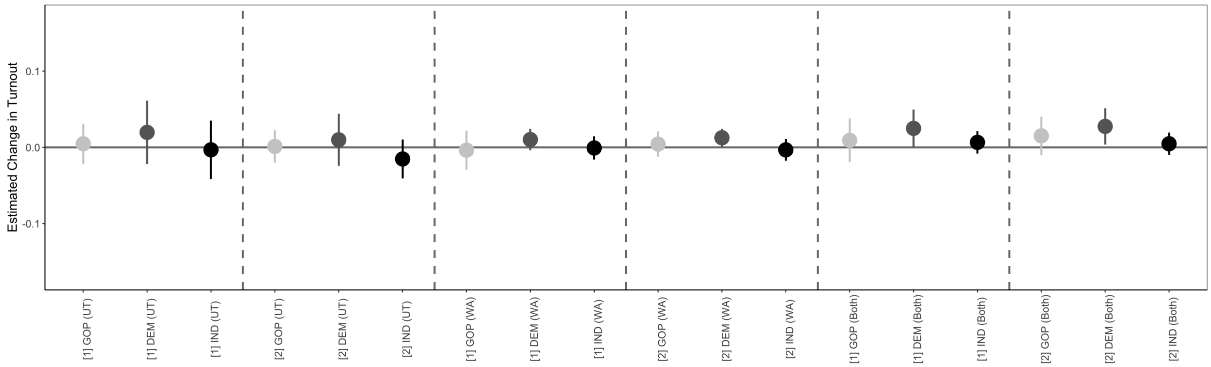

Note: Coefficient plot of additional model specifications in the voter file sample. Points are coefficient estimates and bars are 95% confidence intervals. Model 1 includes individual and year fixed effects. Model 2 includes individual fixed effects, year fixed effects, and a quadratic county-specific time trend.

Figure S12 provides additional results from the individual voter file analyses in Utah and

Washington. To do so, we create aggregate turnout rates by partisan groups for each county-election cycle. We then see if the implementation of VBM has an impact on aggregate turnout at the county level among registered Republicans, Democrats, and Independents separately. The results show that, similar to the individual-level analysis, there are small effects that are not statistically different from zero, or from one another across parties.

Figure S12: Turnout By Partisan Group - Aggregated Voter File Analysis

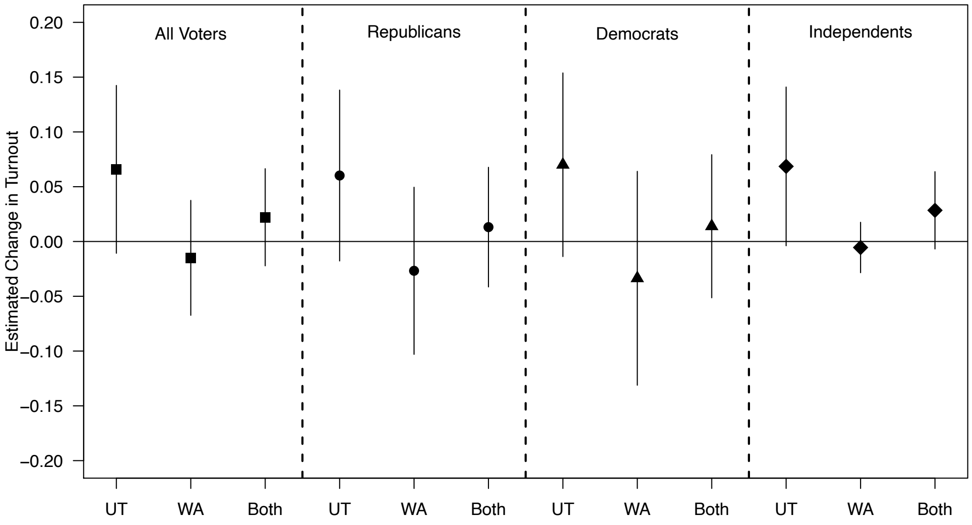

Note: Points are coefficient estimates and bars are 95% confidence intervals. Models include state-by-year and county fixed effects and individual county time trends. Standard errors are clustered at the county level.

9 Testing for Possible Spillover Effects

Figure S13 tests for the possibility that adoption of VBM in a county might have an impact on voting patterns in adjacent counties. Given that changes in the voting system may also lead to changes in how information regarding the election is distributed, discussed in the media, or impacts the strategies of campaigns and candidates, it might be that these changes spill over to adjacent counties. To test for this, we not only code each county for whether or not it has adopted VBM in a particular election, we also code a separate dichotomous variable for any county that is geographically adjacent to a VBM county. We then include both the VBM and VBM-adjacent variables in the original models used in Figure 2 of the main paper. While we still find effects of VBM on turnout, we find no effects for being a VBM-adjacent county.

Figure S13: Testing for Possible Spillover Effects

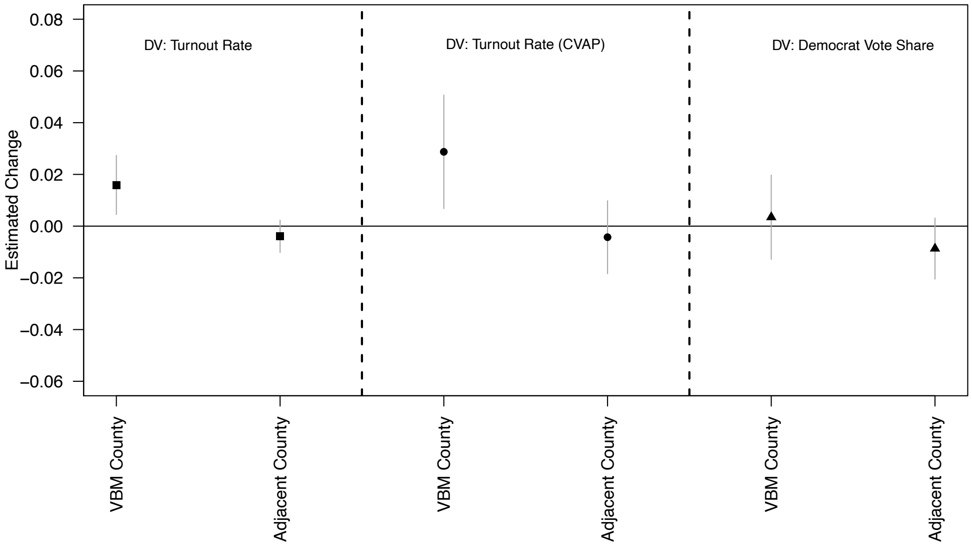

Note: Points are coefficient estimates and bars are 95% confidence intervals. Models include state-by-year and county fixed effects and individual county time trends with heteroskedasticity robust standard errors.

REFERENCES AND NOTES

1. M. Younis, Most Americans favor voting by mail as option in November. *Gallup Report* (2020).

2. G. Elul, S. Freeder, J. M. Grumbach, The effect of mandatory mail ballot elections in california. *Elect. Law J.* **16**, 397–415 (2017).

3. T. Kousser, M. Mullin, Does voting by mail increase participation? Using matching to analyze a natural experiment. *Political Anal.* **15**, 428–445 (2007).

4. A. S. Gerber, G. A. Huber, S. J. Hill, Identifying the effect of all-mail elections on turnout: Staggered reform in the evergreen state. *Polit. Sci. Res. Methods* **1**, 91–116 (2013).

5. A. J. Berinsky, N. Burns, M. W. Traugott, Who votes by mail?: A dynamic model of the individual-level consequences of voting-by-mail systems. *Public Opin. Q.* **65**, 178–197 (2001).

6. R. Larocca, J. S. Klemanski, U.S. state election reform and turnout in presidential elections. *State Pol. Pol. Q.* **11**, 76–101 (2011).

7. D. B. Magleby, Participation in mail ballot elections. *West. Pol. Q.* **40**, 79–91 (1987).

8. A. Menger, R. M. Stein, G. Vonnahme, Turnout effects from vote by mail elections, *Conference on Election Administration and Reform* (2015).

9. R. M. Stein, A. Menger, G. Vonnahme, The impact of vote by mail on the cost and performance of elections in Colorado. *A report prepared for the Pew Charitable Trusts* (2015).

10. A. Menger, R. M. Stein, G. Vonnahme, Reducing the undervote with vote by mail. *Am. Politics Res.* **46**, 1039–1064 (2018).

11. Y. Atsusaka, A. Menger, R. Stein, Compositional effects of vote by mail elections on voter turnout (2019).

12. M. Romero, The california voter experience study: A statewide survey of voter perspectives on vote-by-mail and vote centers. *CCEP Policy Brief Special Series: The California Voter Experience Study*, Issue 3 (2017).

13. M. Romero, Disparities in California's vote-by-mail use changing demographic composition: 2002–2012. *California Civic Engagement Project*, Issue Brief #1 (2014).

14. R. Epstein, S. Saul, Does vote-by-mail favor Democrats? No. it's a false argument by Trump. *The New York Times* (2020).

15. R. M. Alvarez, D. Beckett, C. Stewart III, Voting technology, vote-by-mail, and residual votes in california, 1990–2010. *Polit. Res. Q.* **66**, 658–670 (2013).

16. J. D. Angrist, J.-S. Pischke, *Mostly Harmless Econometrics: An Empiricist's Companion* (Princeton Univ. Press, 2008).

17. J. E. Leighley, J. Nagler, *Who Votes Now?: Demographics, Issues, Inequality, and Turnout in the United States* (Princeton Univ. Press, 2013).

18. J. Kropko, R. Kubinec, Interpretation and identification of within-unit and cross-sectional variation in panel data models. *PLOS ONE* **15**, e0231349 (2020).

19. A. Goodman-Bacon, Difference-in-differences with variation in treatment timing, Tech. rep., National Bureau of Economic Research (2018).

20. C. Wing, K. Simon, R. A. Bello-Gomez, Designing difference in difference studies: Best practices for public health policy research. *Annu. Rev. Public Health* **39**, 453–469 (2018).

21. A. M. Dynes, J. B. Holbein, Noisy retrospection: The effect of party control on policy outcomes. *Am. Polit. Sci. Rev.* **114**, 237–257 (2020).

22. J. D. Angrist, J.-S. Pischke, *Mastering 'metrics: The Path from Cause to Effect* (Princeton Univ. Press, 2014).

23. D. Lakens, A. M. Scheel, P. M. Isager, Equivalence testing for psychological research: A tutorial. *Adv. Methods Pract. Psychol. Sci.* **1**, 259–269 (2018).

24. E. Hartman, F. D. Hidalgo, An equivalence approach to balance and placebo tests. *Am. J. Polit. Sci.* **62**, 1000–1013 (2018).

25. A. Gelman, J. Hill, *Data Analysis Using Regression and Multilevel/Hierarchical Models* (Cambridge Univ. Press, 2006).

26. J. M. Wooldridge, *Econometric Analysis of Cross Section and Panel Data* (MIT Press, 2010).

27. L.-f. Lee, J. Yu, A spatial dynamic panel data model with both time and individual fixed effects. *Economet. Theor.* **26**, 564–597 (2010).

28. H. F. Ladd, C. T. Clotfelter, J. B. Holbein, The growing segmentation of the charter school sector in north carolina. *Educ. Finance Policy* **12**, 536–563 (2017).

29. J. B. Holbein, D. S. Hillygus, M. A. Lenard, C. Gibson-Davis, D. V. Hill, The development of students' engagement in school, community and democracy. *Br. J. Polit. Sci.*, 1–19 (2016).

30. R. M. Alvarez, J. Nagler, Analysis of crossover and strategic voting (1997).

31. G. D. Wekkin, Why crossover voters are not "mischievous voters": The segmented partisanship hypothesis. *Am. Polit. Quart.* **19**, 229–247 (1991).

32. J. B. Holbein, D. S. Hillygus, Making young voters: The impact of preregistration on youth turnout. *Am. J. Polit. Sci.* **60**, 364–382 (2016).

33. L. Keele, W. Minozzi, How much is Minnesota like Wisconsin? Assumptions and counterfactuals in causal inference with observational data. *Polit. Anal.* **21**, 193–216 (2013).

34. D. P. Green, M. C. McGrath, P. M. Aronow, Field experiments and the study of voter turnout. *J. Elect. Publ. Opin. Part.* **23**, 27–48 (2013).

35. B. C. Burden, D. T. Canon, K. R. Mayer, D. P. Moynihan, Election laws, mobilization, and turnout: The unanticipated consequences of election reform. *Am. J. Polit. Sci.* **58**, 95–109 (2014).

36. J. B. Holbein, D. S. Hillygus, *Making Young Voters: Converting Civic Attitudes into Civic Action* (Cambridge Univ. Press, 2020).

37. S. Ansolabehere, E. Hersh, Who really votes?, in *Facing the Challenge of Democracy: Explorations in the Analysis of Public Opinion and Political Participation*, P. M. Sniderman, B. Highton, Eds. (Princeton Univ. Press, 2011), pp. 267–291.

38. J. Citrin, E. Schickler, J. Sides, What if everyone voted? simulating the impact of increased turnout in senate elections. *Am. J. Polit. Sci.* **47**, 75–90 (2003).

39. C. Stewart, Adding up the costs and benefits of voting by mail. *Elect. Law J.* **10**, 297–301 (2011).

40. C. H. Stewart III, Losing votes by mail. *NYUJ Legis. Publ. Policy* **13**, 573–601 (2010).

41. P. L. Southwell, J. Burchett, Survey of vote-by-mail senate election in the state of Oregon. *PS: Polit. Sci. Polit.* **30**, 53–57 (1997).

42. P. L. Southwell, A panacea for voter fatigue? Vote by mail in the state of Oregon. *J. Polit. Mil. Sociol.* **37**, 195–203 (2009).

43. P. L. Southwell, J. I. Burchett, The effect of all-mail elections on voter turnout. *Am. Polit. Quart.* **28**, 72–79 (2000).

44. R. M. Stein, Introduction: Early voting. *Public Opin. Q.* **62**, 57–69 (1998).

45. B. Haas, D. B. Quine, C. E. Woodman, V. Guyett, Vote by mail envelope that protects privacy of voter's signature. US Patent 7766233 (2010).

46. B. Haas, Vote by mail system that allows voters to verify their votes. US Patent 7621450 (2009).

47. M. Venkataraman, W. Henry, A. Shrestha, A. M. Lagneaux, S. M. Dearing, R. E. Garey, S. N. Dunn, Cryptographically tracked and secured vote by mail system. US Patent App. 16101248 (2019).

48. B. Brucker, H. Patria, B. Clubb, L. Wright, B. A. Pittman, D. S. Nelson Jr., R. L. Leonard Jr., B. W. Borne, B. Barrett, J. Wagoner, G. Lemmon, J. Elder, D. Heffernan, Integrated vote by mail processing system. US Patent 7240835 (2007).

49. M. J. Hanmer, M. W. Traugott, The impact of voting by mail on voter behavior. *Am. Polit. Res.* **32**, 375–405 (2004).

50. K. Arceneaux, T. Kousser, M. Mullin, Get out the vote-by-mail? A randomized field experiment testing the effect of mobilization in traditional and vote-by-mail precincts. *Polit. Res. Q.* **65**, 882–894 (2012).

51. N. W. Monroe, D. E. Sylvester, Who converts to vote-by-mail? Evidence from a field experiment. *Elect. Law J.* **10**, 15–35 (2011).

52. K. Smith, D. E. Sylvester, Is it the message or the person? Lessons from a field experiment about who converts to permanent vote by mail. *Elect. Law J.* **12**, 243–260 (2013).

53. H. J. G. Hassell, Teaching voters new tricks: The effect of partisan absentee vote-by-mail get-out-the-vote efforts. *Res. Polit.* **4**, 2053168017694806 (2017).

54. J. Reedy, J. Gastil, P. Moy, From the secret ballot to the public vote: Examining voters' experience of political discussion in vote-by-mail elections. *Polit. Commun.* **33**, 39–58 (2016).

55. E. B. Moreton Jr., Voting by mail. *S. Cal. L. Rev.* **58**, 1261 (1985).

56. P. Gronke, EVIC Director, Ballot integrity and voting by mail: The oregon experience. Report prepared for the National Commission on Election Reform [Online] (2005): http://www.sos.state.or.us/executive/CarterBaker.pdf [accessed 9 October 2006].

57. P. Gronke, P. A. Miller, Annual Meeting of the American Political Science Association (2007).

58. P. Gronke, P. Miller, Voting by mail and turnout in Oregon: Revisiting Southwell and Burchett. *Am. Pol. Res.* **40**, 976–997 (2012).

59. S. Richey, Voting by mail: Turnout and institutional reform in Oregon. *Soc. Sci. Quart.* **89**, 902–915 (2008).

60. P. L. Southwell, Five years later: A re-assessment of Oregon's vote by mail electoral process. *PS: Polit. Sci. Polit.* **37**, 89–93 (2004).

61. P. L. Southwell, J. Burchett, Vote-by-mail in the state of Oregon. *Willamette L. Rev.* **34**, 345 (1998).

62. J. A. Karp, S. A. Banducci, Going postal: How all-mail elections influence turnout. *Polit. Behav.* **22**, 223–239 (2000).

63. P. L. Southwell, Voting behavior in vote-by-mail elections. *Anal. Soc. Issues Public Policy* **10**, 106–115 (2010).

64. P. Southwell, J. Burchett, Does changing the rules change the players? The effect of all-mail elections on the composition of the electorate. *Soc. Sci. Quart.* **81**, 837–845 (2000).

65. S. Richey, Who votes alone? The impact of voting by mail on political discussion. *Aust. J. Polit. Sci.* **40**, 435–442 (2005).

66. S. M. Sled, "It's in the mail: The effect of vote by mail balloting on voter turnout and policy outcomes in us elections," thesis, Massachusetts Institute of Technology (2008).

67. J. Szewczyk, Does vote-by-mail cause voters to gather information about politics?, Tech. rep., Working Paper (2020); http://jamesszewczyk.com/VBM_Information.pdf.

68. B. Fraga, J. Holbein, Measuring youth and college student voter turnout. *Elect. Stud.* **65**, 102086 (2020).

69. B. Nyhan, C. Skovron, R. Titiunik, Differential registration bias in voter file data: A sensitivity analysis approach. *Am. J. Polit. Sci.* **61**, 744–760 (2017).
